# Tofacitinib Treatment of Refractory Cutaneous Leukocytoclastic Vasculitis: A Case Report

**DOI:** 10.3389/fimmu.2021.695768

**Published:** 2021-06-24

**Authors:** Kai-Jun Zhu, Pei-Dan Yang, Qiang Xu

**Affiliations:** ^1^ Department of Rheumatology, The First Affiliated Hospital of Guangzhou University of Chinese Medicine, Guangzhou, China; ^2^ Department of Rheumatology, Zhengzhou Second Hospital, Guangzhou, China; ^3^ The First Clinical Medicine School, Guangzhou University of Chinese Medicine, Guangzhou, China

**Keywords:** cutaneous leukocytoclastic vasculitis, tofacitinib, JAK inhibitor, case report, inflammation

## Abstract

**Introduction:**

To date, there is no treatment with proven efficacy for cutaneous leukocytoclastic vasculitis (CLV). Several reports have suggested that CLV responds favorably to corticosteroids, colchicine, nonsteroidal anti-inflammatory drugs (NSAIDs), azathioprine, and hydroxychloroquine (HCQ). To the best of our knowledge, the oral small molecule Janus kinase inhibitor, tofacitinib, plays an important role in the treatment of autoimmune and inflammatory diseases. Therefore, tofacitinib may be a prospective therapy in patients with CLV.

**Case Presentation:**

A 29-year-old woman presented to our hospital with a 5-year history of symmetric skin lesions mainly affecting both lower extremities. The results for anti-neutrophil cytoplasmic antibodies (ANCA), anti-extracted nuclear antigens (ENA) autoantibodies, anti-double-stranded deoxyribonucleic acid (dsDNA) antibodies, and antinuclear antibodies (ANA) were all negative. The definite diagnosis of CLV was determined by a skin biopsy. However, the patient exhibited a poor response to prednisone, HCQ, methotrexate, colchicine, azathioprine, and tripterygium wilfordii polyglycoside tablets (TGTs) treatments. She was then treated with oral tofacitinib (5 mg twice daily) and oral prednisone (25 mg daily).

**Outcomes:**

Her skin lesions gradually improved over a period of 4 weeks. Two months later, the skin ulcers completely resolved. No evidence of recurrence of skin ulcers was observed during a 6-month follow-up.

**Conclusion:**

We present the first case of a female patient receiving short-term tofacitinib therapy for refractory CLV. Tofacitinib may be a promising oral alternative for patients with CLV. However, its efficacy and safety require further appraisal through clinical trials.

## Introduction

Cutaneous leukocytoclastic vasculitis (CLV) is a small-vessel hypersensitivity vasculitis (1), characterized by skin lesions, fever, general discomfort, and arthralgia (2). The feature of the skin lesions is palpable macular papules or purpura, most of which symmetrically affect the lower extremities and buttocks (3). CLV may involve several systems such as the kidney, joints, gastrointestinal tract, and brain tissue besides skin damage (4). The histopathological features of CLV are massive neutrophil infiltration, nuclear fragmentation, fibrinoid necrosis of the vessel wall, and erythrocyte exosmosis (5). A retrospective study conducted in Minnesota indicated that the incidence of CLV was 4.5 per 100000 person-years, and there was no significant difference between males and females (6). Evidence demonstrated that people with drug allergies, pathogen infections, coagulation system abnormalities, connective tissue disease, and malignant tumors were more prone to develop CLV (7).

The specific pathogenesis of CLV remains unclear. One possible explanation is that the deposition of a specific circulating immune complex could activate the complement cascade, accumulate polymorphonuclear cells, release lysosomal enzymes, and eventually cause damage to the vascular wall (8). Several studies indicated that anti-neutrophil cytoplasmic antibodies (ANCA), inflammatory mediators, and adhesion molecules may also play an important role in the pathogenesis of CLV (9). Corticosteroids, nonsteroidal anti-inflammatory drugs (NSAIDs), colchicine, azathioprine, cyclophosphamide, antihistamines, antimalarials, and hydroxychloroquine (HCQ) have been reported to be effective treatments in several patients with recurrent CLV (10). It was apparent that long-term use of the medium- to high-dose corticosteroids, colchicine, or azathioprine in the treatment of CLV in adults had potential debilitating and fatal side effects (11).

Tofacitinib is an oral Janus kinase (JAK) 3/1 inhibitor that has been approved for the treatment of rheumatoid arthritis (RA) in adults (12). Tofacitinib may suppress inflammatory response by interfering with inflammatory cytokine signaling, and its effectiveness is similar to that of immunosuppressants (13). Although tofacitinib therapy is a suitable alternative to autoimmune and inflammatory diseases (12), its effectiveness in treating CLV has not been reported. Therefore, we herein describe the first case with a 5-year recalcitrant CLV. After treatment failure with prednisone, colchicine, azathioprine, HCQ, tripterygium wilfordii polyglycoside tablets (TGTs), and methotrexate (MTX), the patient achieved a complete recovery with the JAK inhibitor tofacitinib treatment.

## Case Presentation

This was a retrospective investigation of a case with CLV in the First Affiliated Hospital of Guangzhou University of Chinese Medicine. The work described was approved by the Local Ethics Committee, and the patient provided written informed consent. This study was carried out following the Declaration of Helsinki.

In October 2020, a 29-year-old Chinese woman presented to our outpatient department with skin ulcers of the lower limbs and was admitted to our hospital. The patient had a history of recurrent skin ulcers for over 5 years. She had no chronic diseases such as hypertension, diabetes, heart disease, tuberculosis infection, or tumor. According to previous medical reports, ANCA, anti-extracted nuclear antigens (ENA) autoantibodies, anti-double-stranded DNA (dsDNA) antibodies, and antinuclear antibodies (ANA) were negative. Moreover, skin biopsy results revealed that there were a lot of neutrophil infiltration and fibrin deposition in the injured blood vessels, which confirmed the diagnosis of CLV. Unfortunately, we could not trace it back to the original image.

**Figure 1:**
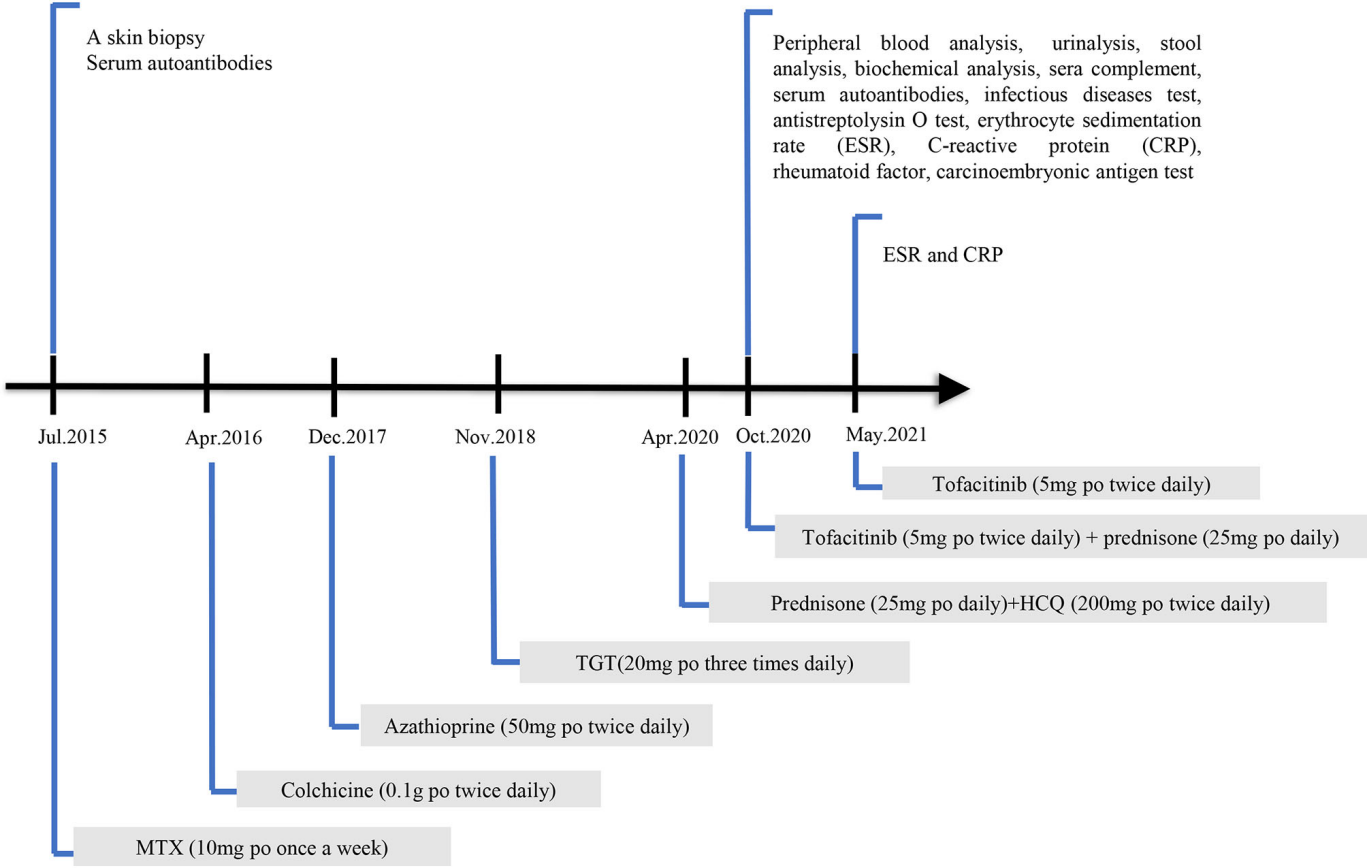
The timeline of laboratory investigations and ongoing treatments.

She was initially on MTX (10 mg per os [po, oral route] once a week) for half a year; however, it was ineffective. Colchicine (0.1 g po three times daily) was discontinued after 1 week due to severe gastrointestinal reactions. The patient was then placed on azathioprine (50 mg po twice daily), which was discontinued after approximately 1 month due to adverse effects of myelosuppression. TGTs (20 mg po three times daily) were prescribed and stopped 2 months later because of abnormal menstruation. Contrarily, thalidomide and mycophenolate mofetil were not recommended because she desired subsequent conception. The patient refused these injections and biological agents were not used. Before visiting our department, she accepted prednisone (25 mg po daily) and HCQ (200 mg po twice daily) as maintenance therapy for half a year. The patient’s condition was improved after prednisone treatment. However, the symptoms recurred when the prednisone treatment was stopped. The details of the laboratory investigations and ongoing treatments are shown in **Figure 1**.

**Figure 2:**
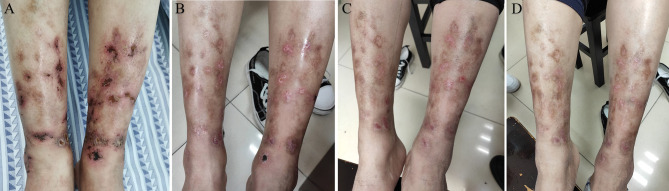
The lower limbs of the patient before and after treatment with tofacitinib. **(A)** The patient’s lower limbs on the initial visit with 2–5 mm visible erythema with some ulceration. **(B)** The lower limbs of the patient after 1 month of tofacitinib administration show an improvement of skin ulcers. **(C)** The patient’s lower limbs after 2 months of tofacitinib treatment reveal complete remission of skin ulcers. **(D)** The patient’s lower limbs did not develop skin ulcers after 6 months of tofacitinib treatment.

Six months previously, skin ulcers developed on the two lower limbs, progressively worsening, and the effect of glucocorticoid treatment was not effective as prior treatments. When the dose of prednisone was reduced by 25 mg daily or lower, the skin ulcers exacerbated. The condition of the skin ulcers is shown in **Figure 2A**. Physical examination in our hospital revealed a symmetrical rash in the calf area, accompanied by visible erythema (size, 2–5 mm). The skin lesions were not tender and swollen. There was no joint pain, Raynaud’s phenomenon, and mucosal involvement. In addition, there were no apparent abnormalities on general examination. Laboratory investigations were as follows: white blood cells: 4.50 × 10^9^/L (neutrophil: 2.54 × 10^9^/L, lymphocyte: 1.48 × 10^9^/L, and eosinophil: 0.19×10^9^/L), hemoglobin: 118 g/L, platelet count: 291 × 10^9^/L, erythrocyte sedimentation rate (ESR): 12 mm/h, C-reactive protein (CRP): 16.3 mg/L, antistreptolysin O test: 160 IU/mL, rheumatoid factor: < 20.0 IU/mL, circulating immune complexes: 2.55 RU/Ml. Anti-myeloperoxidase antibodies, anti-proteinase 3 antibodies, anti-glomerular basement membrane antibodies, anti-dsDNA antibodies, ANCA were all negative. Human immunodeficiency virus, syphilis, hepatitis B, and hepatitis C were also negative. Complement level, urinalysis, and carcinoembryonic antigen test were within normal values.

Given the effectiveness of JAK inhibitor tofacitinib in patients with RA and psoriasis, the patient started a treatment regimen that comprised tofacitinib (5 mg po twice daily) and prednisone (25 mg po daily). One month later, the symptoms improved steadily on this regimen (**Figure 2B**). Complete remission of skin ulcers was achieved after 2 months (**Figure 2C**). The patient stopped taking glucocorticoid treatment, and no evidence of recurrence or side effects was observed throughout the 6 months of follow-up (**Figure 2D**). The follow-up laboratory results indicated significant improvement in inflammatory biomarkers (ESR: 6 mm/h, CRP: 4.5 mg/L). Accordingly, our findings supported the efficacy of tofacitinib in this case investigation.

## Discussion

CLV, a small-vessel hypersensitive vasculitis, remains one of the most challenging diseases in dermatology. Approximately 10% of patients with CLV have experienced an unpleasant recurrence for several years (14). We describe the case of a 29-year-old woman with refractory CLV characterized by symmetric skin ulcers of the lower limbs. She experienced an improvement of skin ulcers when oral tofacitinib was initiated, and complete remission was achieved after 2 months. To the best of our knowledge, our case is the first report of recovery of skin ulcers in adults with CLV treated with tofacitinib.

There is no standardized treatment for severe or recurrent CLV. The evidence of clinical treatment for CLV is limited (15). Clinical researchers suggest that colchicine should be used as the first-line treatment for patients with chronic CLV (15). However, so far, the only randomized controlled study on colchicine in the treatment of CLV has failed to show its efficacy (16). There are also some cases supporting the beneficial effect of dapsone combined with colchicine in the treatment of CLV (17, 18). Alternative therapies such as azathioprine, methotrexate, mycophenolate mofetil, and prednisone can also be considered (19). The condition of the patient with CLV in our case report deteriorated and she showed adverse effects or poor response to conventional treatments such as prednisone, HCQ, MTX, colchicine, and azathioprine. She refused these injections and biological agents were not used. The JAK inhibitors, tofacitinib and baricitinib have shown broad prospects in the treatment of psoriasis, psoriatic arthritis, atopic dermatitis, and alopecia areata (20). Therefore, we decided to use JAK inhibitors to treat this patient. However, baricitinib is more expensive than tofacitinib in China. Considering the patient’s limited economic capacity, we ultimately used tofacitinib for her treatment.

The pathological mechanism of CLV suggests that several immune complexes are deposited on the microvascular wall and a large number of neutrophils accumulated, leading to inflammatory reaction (8, 9). As a small molecule inhibitor of oral JAK agent, tofacitinib primarily inhibited JAK1 and JAK3 (12), disturbed the JAK signaling pathway downstream of an inflammatory cytokine receptor, suppressed most inflammatory cytokines, and blocked active inflammatory cascade reactions (21). We speculated that the mechanism of tofacitinib in the treatment of CLV may involve interference with the signal transduction of one or more proinflammatory cytokines. Adverse effects of tofacitinib including severe infections, gastrointestinal reaction, increased low-density lipoprotein levels decreased neutrophils, and elevated transaminases have previously been reported (22). Although no adverse reactions or infection results were observed in our case, the long-term efficacy and safety of tofacitinib deserve further investigations.

Our study has several limitations. First of all, our findings are based on a single case, and the results may not be universal. Second, this is a case to report the treatment of CLV with tofacitinib, so we cannot determine the molecular mechanism of the treatment of CLV with tofacitinib. We hope to draw a clear conclusion through more relevant studies.

## Conclusion

Tofacitinib may be considered an effective therapy for CLV, especially for individuals who are unwilling to receive injections or those who do not respond to conventional prednisone treatment. However, the long-term efficacy and safety of tofacitinib are unclear, and adverse reactions should be cautious in clinical use. We look forward to encountering more cases to support our conclusions and provide information for the establishment of subsequent clinical trials.

## Data Availability Statement

The original contributions presented in the study are included in the article/supplementary material. Further inquiries can be directed to the corresponding author.

## Ethics Statement

The studies involving human participants were reviewed and approved by First Affiliated Hospital of Guangzhou University of Chinese Medicine. The patients/participants provided their written informed consent to participate in this study. Written informed consent was obtained from the individual(s) for the publication of any potentially identifiable images or data included in this article.

## Author Contributions

KJ-Z and PD-Y collected the data and drafted the manuscript. QX conceived of the study, and participated in designing, writing, reviewing, and revising this manuscript. All authors contributed to the article and approved the submitted version.

## Conflict of Interest

The authors declare that the research was conducted in the absence of any commercial or financial relationships that could be construed as a potential conflict of interest.
